# Diagnostic capability of Pulsar perimetry in pre-perimetric and early glaucoma

**DOI:** 10.1038/s41598-017-03550-x

**Published:** 2017-06-12

**Authors:** Kazunori Hirasawa, Natsumi Takahashi, Kazuhiro Matsumura, Masayuki Kasahara, Nobuyuki Shoji

**Affiliations:** 1 Department of Orthoptics and Visual Science, School of Allied Health Sciences, Kitasato University, Kanagawa, Japan; 2 Department of Ophthalmology, Kitasato University Hospital, Kanagawa, Japan; 3 Department of Ophthalmology, School of Medicine, Kitasato University, Kanagawa, Japan

## Abstract

This study aimed to compare the diagnostic capability of Pulsar perimetry (Pulsar) in pre-perimetric glaucoma (PPG) and early glaucoma (EG) with that of Flicker perimetry (Flicker) and spectral-domain optical conference tomography (SD-OCT). This prospective cross-sectional study included 25 eyes of 25 PPG patients, 35 eyes of 35 EG patients, and 42 eyes of 42 healthy participants. The diagnostic capability using the area under the curve (AUC) of the best parameter and agreement of detectability between structural and functional measurements were compared. For PPG patients, the AUC of Pulsar, Flicker, OCT-disc, and OCT-macular was 0.733, 0.663, 0.842, and 0.780, respectively. The AUC of Flicker was significantly lower than that of OCT-disc (p = 0.016). For EG patients, the AUC of Pulsar, Flicker, OCT-disc, and OCT-macular were 0.851, 0.869, 0.907, and 0.861, respectively. There was no significant difference in AUC among these methods. The agreement between structural and functional measurements expressed by kappa value ranged from −0.16 to 0.07 for PPG and from 0.01 to 0.25 for EG. Although the diagnostic capability of Pulsar in the PPG and EG groups was equal to that of Flicker and SD-OCT, the agreements between structural and functional measurements for both PPG and EG were poor.

## Introduction

Glaucoma is a chronic and progressive optic neuropathy associated with retinal ganglion cell death or dysfunction and with visual field disorder^1^. Although glaucoma is an irreversible disease, visual field progression can be delayed or arrested by reducing intra-ocular pressure to an optimal level^2^. Thus, early assessment using structural and functional ophthalmic measurements is crucial to slow the progression of glaucoma.

Previous studies using conventional standard automated perimetry (SAP) have suggested that structural changes in early glaucoma, including changes to the optic disc^3–10^, the retinal nerve fibre layer (RNFL)^3–10^ and the macular retinal ganglion cell^11–13^, generally occur prior to visual field damage. However, structural damage from glaucoma does not always precede functional damage, and both structural and functional measurements are needed to reliably detect early glaucoma^14, 15^. Although conventional SAP is the clinically accepted method for the diagnosis and assessment of glaucoma, SAP detectability of early glaucomatous visual field damage has been reported to be slightly inferior to the detectability of structural measurements by optical coherence tomography (OCT)^16, 17^ and to functional measurements from non-conventional perimetry, such as short wave length automaed perimetry^18, 19^, flicker perimetry (Flicker)^20, 21^, Moorfields motion displacement test^22, 23^, flicker defined form perimetry^24, 25^, and frequency doubling technology (FDT)^26, 27^.

The Octopus 600 perimeter (Haag-Streit, Koeniz, Switzerland) is based on a thin film transistor LCD and was designed to perform both Pulsar perimetry (Pulsar)^28, 29^ and SAP^30^. Pulsar stimulus was invented in 2000 by Gonzalez-Hernandez and coworkers^28^. Although a prototype device known as the Pulsar T30W test was used in a pilot study, Pulsar was not commercially available until the Octopus 600 perimeter was released in 2013. Pulsar is a flicker stimulus, displaying a ring pattern with different contrast levels in counter phase. Pulsar has been shown to have high detectability for early glaucoma^31–34^. Detectability of early glaucoma using Pulsar has been compared with FDT, rarebit perimetry, the Heidelberg Retina Tomograph II (HRT II), and scanning laser polarimetry (GDx VCC)^31–34^, but there has been no report comparing it to other non-conventional perimetric methods or to spectral-domain OCT (SD-OCT). Nomoto and coworkers^20^ reported that Flicker demonstrates a higher AUC compared to FDT or to SWAP. Thus, there is a need to compare the detectability of Pulsar, Flicker, and SD-OCT. The aim of this study was therefore to assess the diagnostic capability of Pulsar for PPG and EG and to compare this capability with those of Flicker and SD-OCT.

## Results

Three eyes of 3 glaucoma patients and 2 eyes of 2 normal participants were excluded due to failure to meet the inclusion criteria. Thus, 60 eyes of 60 glaucoma patients and 42 eyes of 42 normal participants were analysed. Of the 60 eyes of the 60 glaucoma patients, 25 eyes were classified as pre-perimetric glaucoma (PPG; 24 eyes with normal tension glaucoma and 1 eye with primary open angle glaucoma), and 35 eyes were classified as early glaucoma (EG; 20 eyes with normal tension glaucoma, 11 eyes with primary open angle glaucoma, and 4 eyes with secondary glaucoma). Table 1 shows demographic data of normal participants, PPG patients, and EG patients. Table 2 shows the results of each parameter from Pulsar, Flicker, and SD-OCT in normal participants, PPG patients, and EG patients. Although there was no difference in Flicker results between the control group and the PPG group, there were significant differences in the results from both Pulsar and SD-OCT between the control group and the PPG group.

**Table 1:** Demographics of normal participants and glaucoma patients.

Parameters	(A) Control (n = 43)	(B) PPG (n = 25)	(C) EG (n = 35)	p value		
				(A)–(B)	(A)–(C)	(B)–(C)
Age (years)	55.2 ± 9.7	52.6 ± 13.5	59.4 ± 10.5	1.000	0.277	0.060
IOP (mmHg)	14.0 ± 2.6	15.3 ± 2.5	15.3 ± 2.7	0.618	0.100	1.000
Visual acuity (LogMAR)	−0.20 ± 0.09	−0.15 ± 0.10	−0.16 ± 0.09	0.093	0.143	1.000
Spherical equivalent (D)	−1.97 ± 2.36	−2.12 ± 2.82	−2.54 ± 3.11	1.000	1.000	1.000
SAP MD (dB)	0.75 ± 1.03	0.15 ± 1.13	−1.06 ± 1.01	0.075	<0.001	<0.001
SAP PSD (dB)	1.54 ± 0.26	1.67 ± 0.40	3.52 ± 1.94	1.000	<0.001	<0.001
SAP Fixation loss (%)	3.8 ± 5.7	4.5 ± 7.6	4.5 ± 5.4	1.000	1.000	1.000
SAP False positive (%)	1.6 ± 2.1	2.3 ± 2.3	2.7 ± 2.8	0.758	0.126	1.000
SAP False negative (%)	0.7 ± 1.5	0.2 ± 0.6	1.8 ± 3.5	1.000	0.106	0.023
SAP Test duration (sec)	277.8 ± 31.6	295.1 ± 42.6	321.4 ± 47.1	0.271	<0.001	0.042
SAP Fovea threshold (dB)	37.6 ± 1.6	37.2 ± 2.0	36.9 ± 1.8	1.000	0.244	1.000

PPG = pre-perimetric glaucoma; EG = early glaucoma; IOP = intra-ocular pressure; SAP = standard automated perimetry; MD = mean deviation; PSD = pattern standard deviation.

Data are expressed as mean ± standard deviation.

Statistical comparison was performed with Bonferroni test.

**Table 2:** Comparison of each parameter measured with Pulsar, Flicker, and SD-OCT.

Parameters	(A) Control (n = 43)	(B) PPG (n = 25)	(C) EG (n = 35)	p value		
				(A)–(B)	(A)–(C)	(B)–(C)
**Pulsar perimetry**						
Mean defect (src)	0.3 ± 1.8	1.6 ± 1.7	2.8 ± 2.0	0.018	<0.001	0.043
Square loss variance (src)	1.7 ± 0.7	2.2 ± 0.7	2.7 ± 0.7	0.022	<0.001	0.013
Number of CP < 5%	2.3 ± 3.5	5.0 ± 5.9	8.1 ± 5.8	0.102	<0.001	0.054
Number of CP < 1%	0.5 ± 1.1	1.6 ± 2.8	3.7 ± 3.5	0.300	<0.001	0.005
**Flicker perimetry**						
Mean defect (Hz)	4.2 ± 4.3	7.1 ± 5.8	8.1 ± 4.7	0.055	0.002	1.000
Square loss variance (Hz)	3.8 ± 1.1	4.3 ± 1.0	6.5 ± 2.8	1.000	<0.001	<0.001
Number of CP < 5%	3.0 ± 3.6	3.5 ± 3.8	10.6 ± 7.2	1.000	<0.001	<0.001
Number of CP < 1%	0.3 ± 1.0	1.1 ± 1.8	5.7 ± 6.0	1.000	<0.001	<0.001
**OCT-disc**						
Total thickness (μm)	99.0 ± 8.2	93.4 ± 11.3	81.6 ± 11.4	0.096	<0.001	<0.001
Superior thickness (μm)	119.9 ± 11.8	108.8 ± 17.8	92.8 ± 15.8	0.011	<0.001	<0.001
Temporal thickness (μm)	81.3 ± 13.7	73.2 ± 19.2	73.4 ± 14.5	0.119	0.080	1.000
Inferior thickness (μm)	122.6 ± 12.2	116.8 ± 14.7	96.3 ± 17.7	0.359	<0.001	<0.001
Nasal thickness (μm)	71.9 ± 17.2	75.2 ± 13.2	68.2 ± 15.3	1.000	0.935	0.284
Disc area (mm^2^)	2.15 ± 0.39	2.38 ± 0.55	2.13 ± 0.49	0.148	1.000	0.130
Cup area (mm^2^)	0.75 ± 0.51	1.49 ± 0.77	1.29 ± 0.58	<0.001	0.001	0.589
Rim area (mm^2^)	1.27 ± 0.50	0.81 ± 0.35	0.84 ± 0.33	<0.001	<0.001	1.000
Cup/Disc area ratio	0.33 ± 0.19	0.60 ± 0.21	0.58 ± 0.19	<0.001	<0.001	1.000
Linear CDR	0.54 ± 0.21	0.75 ± 0.20	0.75 ± 0.15	<0.001	<0.001	1.000
Vertical CDR	0.52 ± 0.19	0.74 ± 0.19	0.78 ± 0.16	<0.001	<0.001	1.000
Cup volume (mm^3^)	0.14 ± 0.13	0.45 ± 0.34	0.31 ± 0.28	<0.001	<0.001	1.000
Rim volume (mm^3^)	0.39 ± 0.23	0.16 ± 0.14	0.19 ± 0.14	<0.001	<0.001	1.000
Horizontal DD (mm)	1.55 ± 0.17	1.66 ± 0.22	1.56 ± 0.20	0.064	1.000	0.124
Vertical DD (mm)	1.77 ± 0.16	1.81 ± 0.16	1.75 ± 0.20	1.000	1.000	0.542
**OCT-macular**						
Total mRNFL	36.5 ± 4.0	32.9 ± 3.8	30.1 ± 4.3	0.002	<0.001	0.034
Superior mRNFL	35.2 ± 4.0	31.2 ± 3.3	30.7 ± 5.6	0.002	<0.001	1.000
Inferior mRNFL	37.8 ± 4.6	33.6 ± 60	29.5 ± 5.7	0.007	<0.001	0.015
Total mGCL+	67.4 ± 5.0	64.1 ± 4.3	61.5 ± 5.2	0.028	<0.001	0.133
Superior mGCL+	68.2 ± 5.3	65.2 ± 4.8	63.3 ± 5.4	0.079	<0.001	0.514
Inferior mGCL+	66.2 ± 5.1	63.2 ± 4.6	59.9 ± 6.0	0.084	<0.001	0.052
Total mGCL++	103.7 ± 8.0	97.0 ± 6.0	91.9 ± 8.5	0.003	<0.001	0.044
Superior mGCL++	103.5 ± 8.1	96.3 ± 6.5	93.9 ± 9.5	0.002	<0.001	0.844
Inferior mGCL + +	104.0 ± 8.4	97.4 ± 7.5	89.7 ± 10.9	0.015	<0.001	0.005

PPG = pre-perimetric glaucoma; EG = early glaucoma; CP = corrected probability; OCT = optical coherence tomography; CDR = cup to disc ratio; DD = disc diameter; mRNFL = macular retinal nerve fiber layer; mGCL = macular ganglion cell layer and inner plexiform layer; mGCL +  +  = mRNFL and mGGCL.

Data are expressed as mean ± standard deviation.

Statistical comparison was performed with a Bonferroni test.

**Figure 1:**
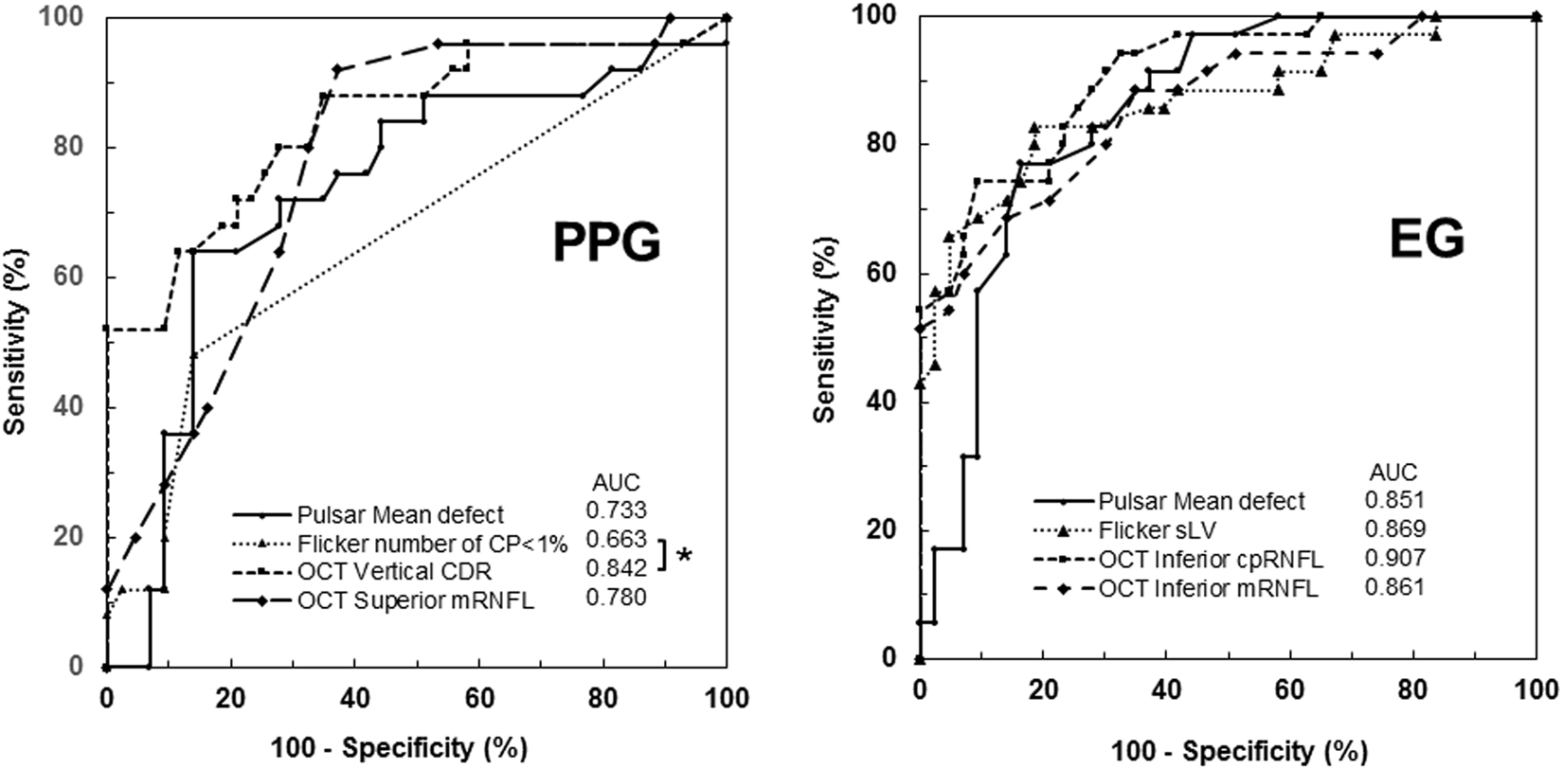
Receiver operating characteristic curve of each device. Area under the curve (AUC) of the best parameters from Pulsar perimetry, Flicker perimetry, optical coherence tomography (OCT) disc measurement, and OCT macular measurement in pre-perimetric glaucoma (PPG) and early glaucoma (EG). The (*) shows significance at p = 0.016 by the DeLong test.

The best parameters for discriminating the control group from the PPG group by Pulsar, Flicker, and SD-OCT (OCT-disc and OCT-macular) were mean defect (AUC = 0.733), number of abnormal points with a p < 0.01 (AUC = 0.663), vertical cup-to-disc ratio (AUC = 0.842), and superior macular RNFL (mRNFL) thickness (AUC = 0.830), respectively. The AUC from the Flicker results was significantly lower than that from the OCT-disc results (p = 0.016), but there was no other significant difference among the three methods. The best parameters for discriminating the control group from the EG group by Pulsar, Flicker, OCT-disc, and OCT-macular were mean defect (AUC = 0.851), square loss variance (AUC = 0.869), inferior circumpapillary RNFL (cpRNFL) thickness (AUC = 0.907), and inferior mRNFL thickness (AUC = 0.861), respectively. There was no significant difference in AUC among the four methods. ROC curves are shown in Fig. 1. The AUC, best cut-off value, sensitivity and specificity, and sensitivity at both 80% and 90% specificity between the control group and the PPG group and between the control group and the EG group are shown in Tables 3 and 4, respectively.

**Table 3:** Results of receiver operating characteristic analysis between control and pre-perimetric glaucoma eyes.

parameters	AUC (SE)	p value	Best cut-off	Se/Sp	Se at 80% Sp	Se at 90% Sp
**Pulsar perimetry**						
Mean defect (src)	***0.733 (0.067)***	<***0.001***	>***1.2***	***64.0***/***86.1***	***64.0***	***36.0***
Square loss variance (src)	0.700 (0.066)	0.002	>1.9	72.0/69.8	32.0	12.4
Number of CP < 5%	0.693 (0.065)	0.003	>0	84.0/51.2	44.8	20.0
Number of CP < 1%	0.680 (0.062)	0.001	>0	60.0/76.7	51.6	25.8
**Flicker perimetry**						
Mean defect (Hz)	0.639 (0.073)	0.057	>9.2	40.0/88.4	40.0	28.0
Square loss variance (Hz)	0.598 (0.072)	0.173	>3.9	60.0/58.1	32.0	24.0
Number of CP < 5%	0.551 (0.073)	0.486	>5	28.0/ 83.7	29.4	8.0
Number of CP < 1%	***0.663 (0.058)***	***0.005***	>***0***	***48.0***/***86.1***	***48.0***	***24.2***
**OCT-disc**						
Total thickness (μm)	0.693 (0.074)	0.009	$\leqq$93	64.0/74.4	50.4	35.1
Superior thickness (μm)	0.700 (0.073)	0.006	$\leqq$103	52.0/88.4	52.0	49.2
Temporal thickness (μm)	0.680 (0.076)	0.018	$\leqq$64	52.0/88.4	52.0	46.4
Inferior thickness (μm)	0.610 (0.076)	0.145	$\leqq$114	52.0/74.4	32.0	32.0
Nasal thickness (μm)	0.570 (0.071)	0.321	<62	92.0/30.2	24.0	12.0
Disc area (mm^2^)	0.622 (0.072)	0.088	>2.45	48.0/81.4	48.0	20.0
Cup area (mm^2^)	0.794 (0.060)	<0.001	>1.19	72.0/83.7	72.0	45.2
Rim area (mm^2^)	0.808 (0.053)	<0.001	$\leqq$1.18	88.0/67.4	62.4	44.0
Cup/Disc area ratio	0.827 (0.055)	<0.001	>0.52	76.0/79.1	72.0	53.2
Linear CDR	0.827 (0.056)	<0.001	>0.72	76.0/79.1	70.4	53.2
Vertical CDR	***0.842 (0.053)***	<***0.001***	>***0.57***	***88.0***/***65.1***	***68.0***	***55.6***
Cup volume (mm^3^)	0.836 (0.057)	<0.001	>0.33	72.0/90.7	76.0	72.0
Rim volume (mm^3^)	0.814 (0.055)	<0.001	$\leqq$0.17	72.0/83.7	72.0	54.4
Horizontal DD (mm)	0.650 (0.070)	0.032	>1.66	52.0/81.4	52.0	16.0
Vertical DD (mm)	0.562 (0.074)	0.405	>1.87	44.0/76.7	30.4	8.6
**OCT-macular**						
Total mRNFL	0.761 (0.060)	<0.001	$\leqq$35	80.0/62.8	48.8	36.8
Superior mRNFL	***0.780 (0.057)***	<***0.001***	$\leqq$ ***34***	***92.0***/***62.8***	***47.7***	***29.2***
Inferior mRNFL	0.719 (0.069)	0.001	$\leqq$32	48.0/86.1	51.5	43.5
Total mGCL+	0.705 (0.065)	0.002	$\leqq$63	48.0/83.7	51.2	33.2
Superior mGCL+	0.669 (0.068)	0.013	$\leqq$69	84.0/44.2	38.9	32.0
Inferior mGCL+	0.672 (0.068)	0.011	$\leqq$63	56.0/74.4	45.6	25.2
Total mGCL++	0.743 (0.060)	<0.001	$\leqq$97	52.0/86.1	52.0	36.6
Superior mGCL++	0.770 (0.056)	<0.001	$\leqq$101	76.0/65.1	76.0	65.1
Inferior mGCL++	0.716 (0.064)	<0.001	$\leqq$100	64.0/72.1	53.6	33.2

AUC = area under the curve; SE = standard erro; Se = sensitivity; Sp = specificity; src; spatial resolution contrast; PPG = pre-perimetric glaucoma; EG = early glaucoma; CP = corrected probability; OCT = optical coherence tomography; CDR = cup to disc ratio; DD = disc diameter; mRNFL = macular retinal nerve fiber layer; mGCL = macular ganglion cell layer and inner plexiform layer; mGCL +  +  = mRNFL and mGCL + .

Data were expressed as mean ± standard deviation.

The highest AUC were expressed by italic bold numbers.

**Table 4:** Results of receiver operating characteristic analysis between control and early glaucoma eyes.

parameters	AUC (SE)	p value	Best cut-off	Se/Sp	Se at 80% Sp	Se at 90% Sp
**Pulsar perimetry**						
Mean defect (src)	***0.851 (0.044)***	<***0.001***	>***1***	***77.1***/***83.7***	***77.1***	***58.0***
Square loss variance (src)	0.851 (0.042)	<0.001	>1.6	94.3/60.5	67.4	57.4
Number of CP < 5%	0.838 (0.044)	<0.001	>0	100/50.2	69.1	50.3
Number of CP < 1%	0.837 (0.044)	<0.001	>0	82.9/76.7	77.5	61.4
**Flicker perimetry**						
Mean defect (Hz)	0.718 (0.057)	<0.001	>5.9	68.6/65.1	42.9	22.9
Square loss variance (Hz)	***0.869 (0.043)***	<***0.001***	>***4.8***	***82.9***/***81.4***	***82.9***	***69.0***
Number of CP < 5%	0.837 (0.048)	<0.001	>4	80.7/81.4	80.7	51.1
Number of CP < 1%	0.837 (0.044)	<0.001	>0	82.9/76.7	77.5	61.1
**OCT-disc**						
Total thickness (μm)	0.891 (0.035)	<0.001	$\leqq$86	65.7/95.4	75.1	70.1
Superior thickness (μm)	0.705 (0.034)	<0.001	$\leqq$110	91.4/79.1	90.3	74.3
Temporal thickness (μm)	0.645 (0.063)	0.022	$\leqq$62	28.6/95.4	40.0	30.3
Inferior thickness (μm)	***0.907 (0.032)***	<***0.001***	$\leqq$ ***105***	***74.3***/***90.7***	***74.3***	***74.3***
Nasal thickness (μm)	0.549 (0.067)	0.466	$\leqq$60	40.0/74.4	24.6	11.6
Disc area (mm^2^)	0.504 (0.068)	0.957	$\leqq$2.31	60.0/25.6	32.3	14.3
Cup area (mm^2^)	0.748 (0.056)	<0.001	>0.54	88.6/53.5	56.0	37.1
Rim area (mm^2^)	0.792 (0.534)	<0.001	$\leqq$1.17	88.6/67.4	64.6	42.9
Cup/Disc area ratio	0.816 (0.049)	<0.001	>0.42	80.0/72.1	70.3	44.6
Linear CDR	0.814 (0.050)	<0.001	>0.60	80.0/72.1	68.6	40.6
Vertical CDR	0.867 (0.044)	<0.001	>0.64	88.6/72.1	68.6	63.7
Cup volume (mm^3^)	0.746 (0.056)	<0.001	>0.09	88.6/53.5	52.0	40.0
Rim volume (mm^3^)	0.776 (0.054)	<0.001	$\leqq$0.27	69.8/37.2	64.6	43.7
Horizontal DD (mm)	0.521 (0.068)	0.761	>1.65	34.3/79.1	32.0	8.6
Vertical DD (mm)	0.515 (0.068)	0.822	$\leqq$1.59	25.7/90.7	27.4	25.7
**OCT-macular**						
Total mRNFL	0.862 (0.041)	<0.001	$\leqq$31	68.6/90.7	72.6	68.9
Superior mRNFL	0.751 (0.058)	<0.001	$\leqq$31	60.0/83.7	62.7	42.1
Inferior mRNFL	***0.861 (0.042)***	<***0.001***	$\leqq$ ***32***	***68.6***/***86.1***	***71.1***	***63.7***
Total mGCL+	0.816 (0.051)	<0.001	$\leqq$63	74.3/83.7	76.6	41.7
Superior mGCL+	0.757 (0.057)	<0.001	$\leqq$66	80.0/67.4	47.4	35.1
Inferior mGCL+	0.803 (0.051)	<0.001	$\leqq$60	62.9/86.1	65.1	48.3
Total mGCL + +	0.855 (0.043)	<0.001	$\leqq$97	77.1/86.1	78.4	54.7
Superior mGCL + +	0.783 (0.053)	<0.001	$\leqq$98	68.6/79.1	66.3	49.4
Inferior mGCL + +	0.854 (0.043)	<0.001	$\leqq$100	85.7/72.1	70.9	61.9

PPG = pre-perimetric glaucoma; EG = early glaucoma; CP = corrected probability; OCT = optical coherence tomography; CDR = cup to disc ratio; DD = disc diameter; mRNFL = macular retinal nerve fiber layer; mGCL = macular ganglion cell layer and inner plexiform layer; mGCL +  +  = mRNFL and mGGCL + .

Data are expressed as mean ± standard deviation.

The highest AUC are expressed by italic bold numbers in each device.

Comparison of sensitivity and specificity values at best cut-off and sensitivity values at 80% and 90% specificity values are shown in Table 5. For the PPG group, sensitivity at best cut-off and at 90% specificity of Pulsar was significantly lower than that of OCT-disc (p < 0.013) and OCT-macular (p < 0.008). However, specificity at best cut-off of Pulsar was significantly higher than that of OCT-disc (p = 0.003) and OCT-macular (p = 0.001). The sensitivity and specificity of Pulsar were equal to or better than that of Flicker. For EG, sensitivity and specificity at best cut-off and sensitivity at 80% specificity of Pulsar were equal to Flicker, OCT-disc, and OCT-macular. However, sensitivity at 90% specificity of Pulsar was significantly lower than that of OCT-disc (p = 0.030).

**Table 5:** Statistical comparison of sensitivity and specificity values at best cut-off and sensitivity values at fixed specificity among each device.

Parameters	Se at best cut-off	Sp at best cut-off	Se at 80% Sp	Se at 90% Sp
**Pre-Perimetric Glaucoma (PPG)**				
Pulsar mean defect/	64.0%/48.0%	86.1%/86.1%	64.0%/48.0%	36.0%/24.2%
Flicker Number of CP < 1%	p = 0.032	p = 0.500	p = 0.003	p = 0.066
Pulsar mean defect/	64.0%/88.0%	86.1%/65.1%	64.0%/68.0%	36.0%/55.6%
OCT-disc Vertical CDR	p = 0.001	p = 0.003	p = 0.292	p = 0.013
Pulsar mean defect/	64.0%/92.0%	86.1%/62.8%	64.0%/47.7%	36.0%/29.2%
OCT-macular Superior mRNFL	p < 0.001	p = 0.001	p = 0.030	p = 0.008
Flicker Number of CP < 1% /	48.0%/88.0%	86.1%/65.1%	48.0%/68.0%	24.2%/55.6%
OCT-disc Vertical CDR	p < 0.001	p = 0.003	p = 0.012	p < 0.001
Flicker Number of CP < 1% /	48.0%/92.0%	86.1%/62.8%	48.0%/47.7%	24.2%/29.2%
OCT-macular Superior mRNFL	p < 0.001	p = 0.002	p = 0.483	p < 0.001
OCT-disc Vertical CDR /	88.0%/92.0%	65.1%/62.8%	68.0%/47.7%	55.6%/29.2%
OCT-macular Superior mRNFL	p = 0.203	p = 0.375	p = 0.011	p = 0.002
**Early glaucoma (EG)**				
Pulsar mean defect /	77.1%/82.9%	83.7%/81.4%	77.1%/82.9%	58.0%/69.0%
Flicker square loss variance	p = 0.185	p = 0.345	p = 0.185	p = 0.101
Pulsar mean defect /	77.1%/74.3%	83.7%/90.7%	77.1%/74.3%	58.0%/74.3%
OCT-disc Inferior Thickness	p = 0.334	p = 0.105	p = 0.334	p = 0.0300
Pulsar mean defect /	77.1%/68.6%	83.7%/86.1%	77.1%/71.1%	58.0%/63.7%
OCT-macular Inferior mRNFL	p = 0.125	p = 0.330	p = 0.197	p = 0.257
Flicker square loss variance/	82.9%/74.3%	81.4%/90.7%	82.9%/74.3%	69.0%/74.3%
OCT-disc Inferior Thickness	p = 0.105	p = 0.059	p = 0.105	p = 0.230
Flicker square loss variance/	82.9%/68.6%	81.4%/86.1%	82.9%/71.1%	69.0%/63.7%
OCT-macular Inferior mRNFL	p = 0.028	p = 0.213	p = 0.052	p = 0.239
OCT-disc Inferior Thickness/	74.3%/68.6%	90.7%/86.1%	74.3%/71.1%	74.3%/63.7%
OCT-macular Inferior mRNFL	p = 0.215	p = 0.217	p = 0.320	p = 0.087

Se = sensitivity; Sp = specificity; CP = corrected probability; OCT = optical coherence tomography; CDR = cup to disc ratio; mRNFL = macular retinal nerve fiber layer.

Data were analyzed by χ^2^ test.

**Figure 2:**
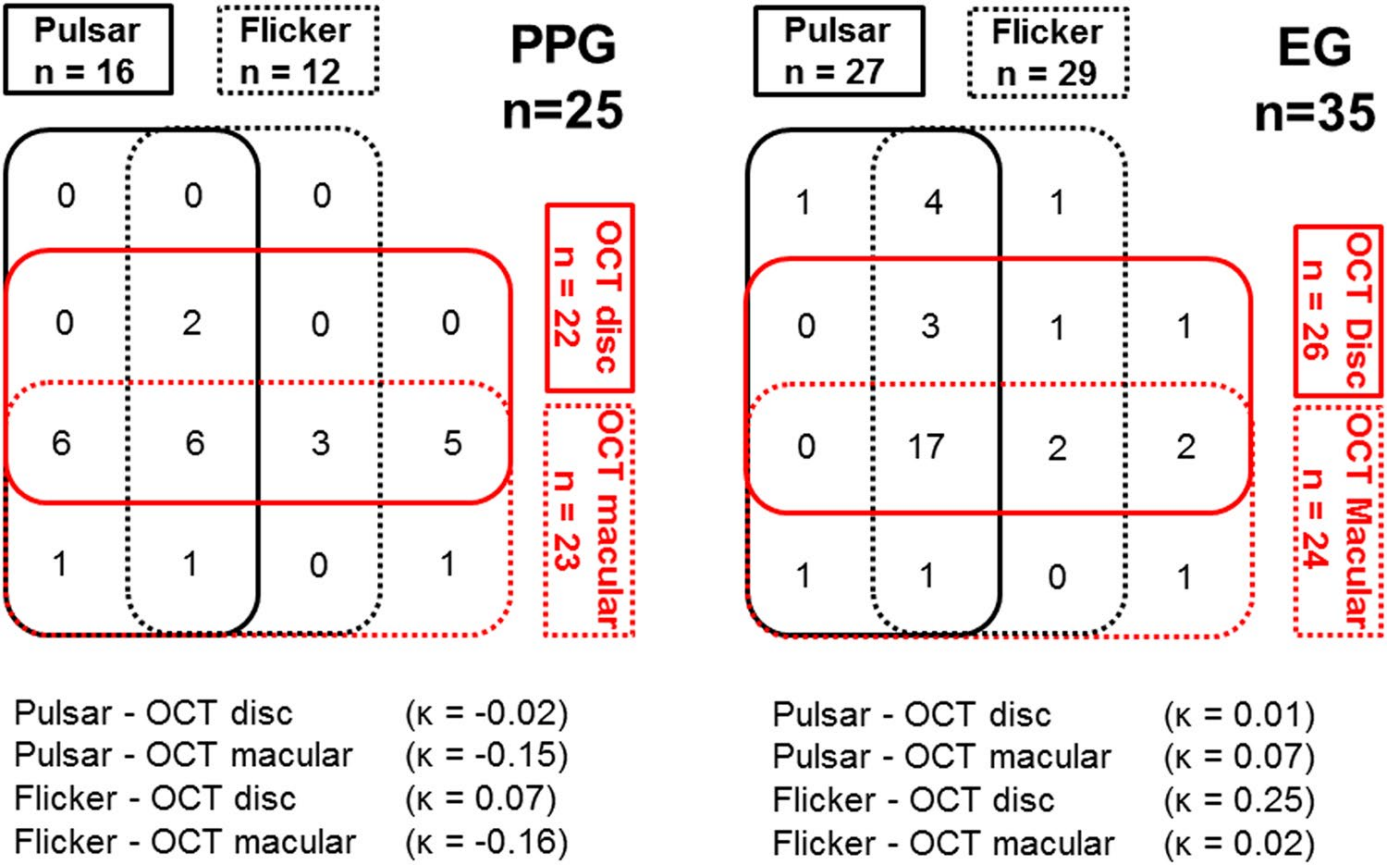
Venn diagram of the eyes detected as abnormality in each device. Abnormality is based on the cut-off value of the best parameter of Pulsar perimetry, Flicker perimetry, optical coherence tomography (OCT) disc measurement, and OCT macular measurement. The agreement values between structural and functional measurements are expressed by kappa coefficients under the Venn diagram.

Figure 2 shows a Venn diagram of Pulsar, Flicker, OCT-disc and OCT-macular parameters, showing the agreement between structural and functional measurements. Abnormality was based on the cut-off value of the best parameter. There were no patients with normal results for all devices. Six of the 25 PPG eyes (24%) and 17 of the 35 EG eyes (48.6%) showed abnormal results from all four methods. However, PPG and EG were detected with 100% sensitivity by functional or structural measurement. The agreement between structural and functional measurements expressed by kappa values ranged from −0.16 to 0.07 in the PPG group and from 0.01 to 0.25 in the EG group.

Table 6 shows the statistical comparison of the test duration and reliability index for both the false positive (FP) and false negative (FN) response rates between Pulsar and Flicker perimetry. The reliability index of Flicker was significantly worse than that of Pulsar in the control group, the PPG group, and the EG group (p < 0.001). Test duration of Pulsar was significantly shorter than that of Flicker in the control group (p < 0.001), the PPG group (p < 0.001), and the EG group (p < 0.001).

**Table 6:** Statistical comparison of reliability indices and test duration between Pulsar and Flicker perimetry.

parameters	Pulsar	Flicker	p value
**Normal participants (control)**			
False positive rate (%)	0.0 [0.0–0.0]	0.0 [0.0–0.0]	1.000
False negative rate (%)	0.0 [0.0–0.0]	6.3 [0.0–7.1]	<0.001
Test duration (second)	162.6 ± 17.2	211.0 ± 50.9	<0.001
**Pre-perimetric glaucoma (PPG)**			
False positive rate (%)	0.0 [0.0–0.0]	11.1 [0.0–14.3]	<0.001
False negative rate (%)	0.0 [0.0–0.0]	0.0 [0.0–0.0]	1.000
Test duration (second)	172.8 ± 26.0	218.0 ± 21.3	<0.001
**Early glaucoma (EG)**			
False positive rate (%)	0.0 [0.0–0.0]	11.1 [0.0–14.3]	<0.001
False negative rate (%)	0.0 [0.0–0.0]	0.0 [0.0–0.0]	1.000
Test duration (second)	176.2 ± 11.3	213.1 ± 21.4	<0.001

False positive and negative are expressed as median value [interquartile range] and analyzed by Wilcoxon signed-rank test.

Test duration are expressed as mean ± standard deviation and analyzed by paired t-test.

## Discussion

In the current study, we found that the diagnostic capability of Pulsar was equal to Flicker and SD-OCT for both PPG and EG patients. However, the agreement of detectability of structural and functional measurements was poor, and structural measurements appeared to be more sensitive than functional measurements in PPG patients. In contrast, functional measurements using Pulsar were equal to structural measurement in EG patients.

To date, many studies have reported on the diagnostic capability for PPG using specific functional measurements with methods such as FDT (AUC = 0.666 to 0.802)^20, 35–37^, Flicker (AUC = 0.800)^20^, SWAP (AUC = 0.660 to 0.704)^20, 37^, and Pulsar (AUC = 0.733)^31^. Multiple studies have also investigated the diagnostic capability for PPG using structural measurements with methods such as OCT (AUC = 0.527 to 0.938)^38–40^, HRT (AUC = 0.740 to 0.914)^39, 40^, and GDx (AUC = 0.688 to 0.894)^39^. Although it is difficult to make a direct statistical comparison between the AUC results of the current study and those of previous studies because of differences in sample size and characteristics, we report an AUC for PPG of 0.733 using Pulsar, similar to the results of previous studies on specific functional measurements^20, 31, 35–37^. Although the AUC from Pulsar did not differ from other devices in the current study, the AUCs from specific functional measurements were slightly lower than those that have been reported by previous studies. When sensitivity values at the best cut-off or at fixed specificity were compared for each device, the structural measurements from OCT appeared more sensitive than the specific functional measurements reported by the current study and by a previous study^31^. This might be because PPG was determined by clinical structural assessment of the optic disc shape based on the general mechanism of pathogenesis^1, 41, 42^. The best parameter for discriminating between the control group from the PPG patients was cup shape, not cpRNFL thickness. Indeed, the diagnostic capability of structural measurements of the optic nerve head is better than cpRNFL thickness for classifying PPG^43, 44^.

For EG, the diagnostic capabilities using AUC of both Pulsar and Flicker were equal to those determined from SD-OCT. This is in agreement with a previous study reporting that the diagnostic capability of Pulsar using AUC was equal to FDT, HRT II, and GDx VCC^31^. Functional measurements appear to be more sensitive for EG diagnosis than structural measurements in both a previous study^31^ and the current study; however, we report the opposite for PPG diagnosis. This might be due to the fact that all EG patients had abnormal SAP results corresponding with Anderson-Patella criteria^45^ in addition to a glaucomatous optic nerve head. Additionally, as glaucoma progresses from PPG, superior or inferior RNFL thinning occurs along with changes to the optic nerve head. Thus, it could be reasonably expected that functional measurements are more sensitive than structural measurements for EG diagnosis.

Although the sensitivity for PPG at best cut-off of Pulsar was lower than the sensitivity of OCT, specificity at best cut-off was higher than OCT. However, both sensitivity and specificity for EG at best cut-off with Pulsar was equal to OCT. The agreement between structural and specific functional measurements in both PPG and EG was poor. However, PPG and EG were detected with either structural or specific functional measurements with 100% sensitivity (Fig. 2). There was no method able to accurately detect glaucoma using only one parameter. Based on the current study, the combination of OCT for structural measurement and Pulsar for selective functional measurement should be recommended to reliably diagnose PPG and EG.

Reliability indices of FP and FN for Flicker were worse than Pulsar, and an especially high FP rate was demonstrated for Flicker in both PPG and EG. Flicker fusion frequency was measured at each test point with a fixed high contrast stimulus of 0 dB, while contrast sensitivity was measured with a fixed temporal frequency of 10 Hz for Pulsar. Eyes with PPG or EG, such as those investigated in this study, can still respond to Flicker stimuli, although with decreased sensitivity. Flicker is difficult for PPG or EG patients to accurately respond to, as the flickering target is close to threshold at slightly decreased sensitivity regions. It was reported that threshold variability increases even the SAP measurements at slightly decreased sensitivity region^46^.

The test duration of Pulsar was shorter than that of Flicker despite the use of the same tendency oriented perimetry (TOP) algorithm. This may be due to the difference in number of test points between Pulsar and Flicker. The 32 P test point of Pulsar is similar to the original Octopus 32 test point program with a 6-degree interval, but the 4 points at the superior and inferior were each removed. In contrast, Flicker was measured with the original Octopus 32 test point program. Another reason for the difference in stimulus presentation time might be that a presentation time of 500 msec was applied for Pulsar, but only a 1 sec presentation time was applied for Flicker.

Pulsar and Flicker each have several advantages and disadvantages. Pulsar may have the advantage of ease of use and less fatigue compared with Flicker because it demonstrated good reliability indices and a shorter test duration in the current study, and a previous study reported that Pulsar is not associated with a learning effect^47^, but that Flicker is^48^. However, it was reported that Pulsar was affected with intraocular straylight as well as SAP^49^. In contrast, Flicker didn’t affect ocular media opacity^50^. Thus, Pulsar may have the disadvantage of robustness to media opacities compared with Flicker.

The current study’s main limitation was that the rate of glaucoma type was different between PPG and EG, and PPG patients in particular had almost normal tension glaucoma. Further studies will therefore be required to confirm our results.

In conclusion, the diagnostic capability of Pulsar for PPG and EG was equal to that of Flicker and OCT. However, the agreement between structural and functional measurements for PPG and EG was poor. The structural measurements from OCT were more sensitive than the specific functional measurements from Pulsar for PPG, while specific functional measurements by Pulsar were more sensitive than the structural measurement by OCT for EG. Therefore, a combination of structural and functional measurements is recommended to reliably diagnose early glaucoma.

## Methods

This prospective cross-sectional study was reviewed and approved by the Kitasato University Hospital Ethics Committee (no. B14-129). All study conduct adhered to the tenets of the Declaration of Helsinki, and all study subjects provided written informed consent. This study was registered in the UMIN Clinical Trials Registry (http://www.umin.ac.jp/) under the unique trial number UMIN000016055.

### Study participants

This study included sixty-three eyes of 63 open angle glaucoma patients who visited the Kitasato University Hospital Glaucoma Service between November 2014 and May 2016 and who had previous SAP results from a Humphrey Field Analyzer (HFA) 24–2 or 30–2 Swedish Interactive Threshold Algorithm (SITA) Standard better than a mean deviation of −3 dB. Additionally, the control group consisted of 45 eyes of 45 normal volunteers from a population of Kitasato University Hospital medical staff and Kitasato University staff members who were recruited between January and May 2016 and who had previous SAP measurements from a HFA 24–2 or 30–2 SITA Standard at least 2 times within one year. The diagnosis of glaucoma was determined via a fundus examination using slit-lamp indirect ophthalmoscopy and 90-diopter lens by one of three glaucoma specialists (MK, KM, or NS) and based on previous SAP results. All glaucoma patients and normal participants underwent a comprehensive ophthalmic examination, including noncycloplegic refraction testing, visual acuity testing at 5 meters using a Landolt ring chart, intraocular pressure measurement, ocular axial length measurement, and slit-lamp and fundus examination by a glaucoma specialist (MK, KM, or NS). Glaucoma patients were included in this study if they had a corrected visual acuity of 20/20 or better, cylindrical power of −1.50 diopter or less, and spherical equivalent of −8.00 to +5.00 diopter. These criteria were also applied to normal participants, with the added criteria of an intraocular pressure of 21 mmHg or less, a normal optic disc appearance, and no ophthalmic diseases in the absence of refractive error.

After comprehensive ophthalmic examination, all glaucoma patients and normal participants underwent an initial SAP measurement. This SAP measurement was performed with an HFA (Carl Zeiss Meditec, Dublin, CA) 24–2 or 30–2 SITA Standard. SAP results were considered reliable if the fixation loss was <20%, the false positive rate was <15%, and the false negative rate was <33%.

### Early glaucoma patients

After SAP measurement, glaucoma patients were classified as EG if they showed structural glaucoma changes such as rim thinning, notching, and nerve fibre layer thinning or defects, and if they showed abnormal SAP results corresponding with Anderson-Patella criteria^45^.

### Pre-perimetric glaucoma patients

After SAP measurement, patients were classified as PPG if they showed the abovementioned structural glaucoma changes in the absence of abnormal SAP results. After SAP measurements, all patients and normal participants underwent Pulsar, Flicker and SD-OCT in random order.

### Pulsar perimetry

Pulsar perimetry was performed using the Octopus 600 perimeter 32 P TOP algorithm. The 32 P test point is similar to the original Octopus 32 test point program with a 6 degree interval, but the 4 points at the superior and inferior were each removed because of limitations of the angle of field of the monitor. The stimulus consisted of a circular, sinusoidal, 5-degree diameter grating pattern that was presented for 500 msec. The stimulus underwent a counter phase pulse motion at 10 Hz, in which both spatial resolution (from 0.5 to 6.3 cycle/degree on a 12-step log scale) and contrast (from 3 to 100% on a 32-step log scale) were simultaneously modified. Threshold sensitivity is expressed in spatial resolution contrast units (src). Refractive error was corrected to distance by inserting trial lenses with the spherical equivalent correction into the eye piece. The presentation ratios of FP and FN catch trials were configured to 10% of the total number of stimuli presented for Octopus 600 testing reliability, which corresponds with those of the HFA SAP performed with the SITA protocol.

### Flicker perimetry

Flicker perimetry was performed using the Octopus 311 perimeter (Haag-Streit, Koeniz, Switzerland) 32 TOP algorithm. The stimulus consisted of a Goldmann size III (0.43 degree) target with a luminance of 1527 cd/m^2^ (4800 apostilbs) that was presented for 1 sec. The flicker targets were presented under a supra-threshold condition with a background luminance of 10 cd/m^2^ (31.5 apostilbs), and critical flicker frequency values were evaluated at each test point. Threshold sensitivity is expressed in critical flicker frequency (Hz). The presentation ratios of FP and FN catch trials were configured to 10% of the total number of stimuli presented for Octopus 311 testing reliability, which corresponds with those of HFA SAP performed with the SITA protocol.

### SD-OCT

SD-OCT imaging was performed using 3D OCT-2000 version 8.1.1 (Topcon, Tokyo, Japan) in the 3D optic disc horizontal raster scan mode (OCT-disc) with a 512 × 128 scan resolution and 6 × 6 mm scan area and in the 3D macular vertical raster scan mode (OCT-macular) with a 512 × 128 scan resolution and 7 × 7 mm scan area. This device operates at a speed of 50,000 A-scans per second and has a depth and lateral resolution of 6 and 20 μm or less, respectively. It requires a pupil size of 2.5 mm or larger for imaging. Ocular magnification was corrected based on Littmann’s formula^51^.

### Outcome measures and exclusion criteria

The main outcome measures were the diagnostic capability of each device using the best cut-off parameter for discriminating between healthy and glaucomatous eyes and the agreement of detectability between structural and functional measurements. The secondary outcome measures were the comparison of reliability indices, including FP and FN, and the test duration between Pulsar and Flicker.

All examinations were performed within a three-month period. The results of the first examination were excluded to avoid learning effects. Right eye results were converted to left eye format for analysis. The exclusion criteria were as follows: fixation loss >20% and false positive rate >15% in HFA measurement; reliability factor >15%, which is the average of the FP and FN rates in Flicker and Pulsar; and image quality index <30 in SD-OCT.

### Statistical analysis

Normality of the data distribution was evaluated using the Shapiro-Wilk test. Test results were compared using either paired t-tests or Wilcoxon signed-rank tests. A Bonferroni test was used to correct for multiple testing. The best cut-off parameter for each device for discrimination between healthy and glaucomatous eyes was decided by the highest AUC based on receiver operating characteristic analysis. The detectability of each device was assessed using the AUC of the best cut-off parameter by the DeLong method. Kappa statistics were calculated to evaluate agreement of detectability between structural and functional measurements. All data were analysed using commercially available SPSS version 22.0 (IBM Japan Ltd, Tokyo, Japan) and MedCalc version 16.1 (MedCalc Software, Ostend, Belgium).

## References

[1] Weinreb RN, Khaw PT (2004). Primary open-angle glaucoma. Lancet.

[2] Garway-Heath DF (2015). Latanoprost for open-angle glaucoma (UKGTS): a randomised, multicentre, placebo-controlled trial. Lancet.

[3] Chauhan BC, McCormick TA, Nicolela MT, LeBlanc RP (2001). Optic disc and visual field changes in a prospective longitudinal study of patients with glaucoma: comparison of scanning laser tomography with conventional perimetry and optic disc photography. Arch Ophthalmol.

[4] Bengtsson B (1990). Optic disc haemorrhages preceding manifest glaucoma. Acta Ophthalmol (Copenh).

[5] Airaksinen PJ, Heijl A (1983). Visual field and retinal nerve fibre layer in early glaucoma after optic disc haemorrhage. Acta Ophthalmol (Copenh).

[6] Sommer A (1991). Clinically detectable nerve fiber atrophy precedes the onset of glaucomatous field loss. Arch Ophthalmol.

[7] Quigley HA, Katz J, Derick RJ, Gilbert D, Sommer A (1992). An evaluation of optic disc and nerve fiber layer examinations in monitoring progression of early glaucoma damage. Ophthalmology.

[8] Tuulonen, A., Lehtola, J. & Airaksinen, P. J. Nerve fiber layer defects with normal visual fields. Do normal optic disc and normal visual field indicate absence of glaucomatous abnormality? *Ophthalmology* **100**, 587–597, discussion 597–588 (1993). 10.1016/s0161-6420(93)31598-8 8493001

[9] Caprioli J, Nouri-Mahdavi K, Law SK, Badala F (2006). Optic disc imaging in perimetrically normal eyes of glaucoma patients with unilateral field loss. Trans Am Ophthalmol Soc.

[10] Zeyen TG, Caprioli J (1993). Progression of disc and field damage in early glaucoma. Arch Ophthalmol.

[11] Tan, O. *et al*. Detection of macular ganglion cell loss in glaucoma by Fourier-domain optical coherence tomography. *Ophthalmology* **116**, 2305–2314.e2301–2302 (2009). 10.1016/j.ophtha.2009.05.025 PMC2787911 19744726

[12] Rolle T, Briamonte C, Curto D, Grignolo FM (2011). Ganglion cell complex and retinal nerve fiber layer measured by fourier-domain optical coherence tomography for early detection of structural damage in patients with preperimetric glaucoma. Clin Ophthalmol.

[13] Nakano N (2011). Macular ganglion cell layer imaging in preperimetric glaucoma with speckle noise-reduced spectral domain optical coherence tomography. Ophthalmology.

[14] Hood DC, Kardon RH (2007). A framework for comparing structural and functional measures of glaucomatous damage. Prog Retin Eye Res.

[15] Tatham AJ, Medeiros FA, Zangwill LM, Weinreb RN (2015). Strategies to improve early diagnosis in glaucoma. Prog Brain Res.

[16] Bagga H, Feuer WJ, Greenfield DS (2006). Detection of psychophysical and structural injury in eyes with glaucomatous optic neuropathy and normal standard automated perimetry. Arch Ophthalmol.

[17] Horn FK (2011). Frequency doubling technique perimetry and spectral domain optical coherence tomography in patients with early glaucoma. Eye (Lond).

[18] Mok KH, Lee VW (2000). Nerve fiber analyzer and short-wavelength automated perimetry in glaucoma suspects: a pilot study. Ophthalmology.

[19] Fogagnolo P, Rossetti L, Ranno S, Ferreras A, Orzalesi N (2008). Short-wavelength automated perimetry and frequency-doubling technology perimetry in glaucoma. Prog Brain Res.

[20] Nomoto H (2009). Detectability of glaucomatous changes using SAP, FDT, flicker perimetry, and OCT. J Glaucoma.

[21] Matsumoto C (2006). Automated flicker perimetry in glaucoma using Octopus 311: a comparative study with the Humphrey Matrix. Acta Ophthalmol Scand.

[22] Dabasia PL, Fidalgo BR, Edgar DF, Garway-Heath DF, Lawrenson JG (2015). Diagnostic Accuracy of Technologies for Glaucoma Case-Finding in a Community Setting. Ophthalmology.

[23] Ong EL (2014). Performance of the Moorfields motion displacement test for identifying eyes with glaucoma. Ophthalmology.

[24] Lamparter J (2012). Structure-function relationship between FDF, FDT, SAP, and scanning laser ophthalmoscopy in glaucoma patients. Invest Ophthalmol Vis Sci.

[25] Horn FK, Tornow RP, Junemann AG, Laemmer R, Kremers J (2014). Perimetric measurements with flicker-defined form stimulation in comparison with conventional perimetry and retinal nerve fiber measurements. Invest Ophthalmol Vis Sci.

[26] Johnson CA, Samuels SJ (1997). Screening for glaucomatous visual field loss with frequency-doubling perimetry. Invest Ophthalmol Vis Sci.

[27] Medeiros FA, Sample PA, Weinreb RN (2004). Frequency doubling technology perimetry abnormalities as predictors of glaucomatous visual field loss. Am J Ophthalmol.

[28] Gonzalez-Hernandez, M., Pareja, R. A., Rodriguez, M. & G de la R, M. Combined spatial resolution and contrast perimetry in normal subjects. *Perimetry Update 2000/2001* (eds Wall M., Mills R. P.) 109–114 (Kugler 2001).

[29] Gonzalez-Hernandez, M., Abreu, A., Sanchez, M. & Gonzalez de la Rosa, M. Combined spatial, contrast and temporal function perimetry in early glaucoma and ocular hypertension. *Perimetry Update 2002/2003* (eds Henson D. B., Wall M.) 247 (Kugler 2004).

[30] Gonzalez de la Rosa M, Gonzalez-Hernandez M, Lozano Lopez V, Perera Sanz D (2007). Topographical spatial summation in glaucoma. Eur J Ophthalmol.

[31] Zeppieri M (2010). Pulsar perimetry in the diagnosis of early glaucoma. Am J Ophthalmol.

[32] Salvetat ML, Zeppieri M, Tosoni C, Parisi L, Brusini P (2010). Non-conventional perimetric methods in the detection of early glaucomatous functional damage. Eye (Lond).

[33] Gonzalez-de-la-Rosa M, Gonzalez-Hernandez M, Aguilar-Estevez J, Diaz-Aleman T, Armas-Plasencia R (2007). [Diagnostic capability of PULSAR, FDT y HRT-II in glaucoma suspects]. Arch Soc Esp Oftalmol.

[34] Gonzalez-Hernandez M, Garcia-Feijoo J, Mendez MS, de la Rosa MG (2004). Combined spatial, contrast, and temporal functions perimetry in mild glaucoma and ocular hypertension. Eur J Ophthalmol.

[35] Choi JA, Lee NY, Park CK (2009). Interpretation of the Humphrey Matrix 24-2 test in the diagnosis of preperimetric glaucoma. Jpn J Ophthalmol.

[36] Mastropasqua L (2006). Humphrey matrix frequency doubling technology perimetry and optical coherence tomography measurement of the retinal nerve fiber layer thickness in both normal and ocular hypertensive subjects. J Glaucoma.

[37] Horn FK, Brenning A, Junemann AG, Lausen B (2007). Glaucoma detection with frequency doubling perimetry and short-wavelength perimetry. J Glaucoma.

[38] Cennamo, G. *et al*. Structure-Functional Parameters in Differentiating Between Patients With Different Degrees of Glaucoma. *J Glaucoma* (2016). 10.1097/IJG.0000000000000491 27483418

[39] Ferreras A (2007). Can frequency-doubling technology and short-wavelength automated perimetries detect visual field defects before standard automated perimetry in patients with preperimetric glaucoma?. J Glaucoma.

[40] Shin HY, Park HY, Jung KI, Park CK (2013). Glaucoma diagnosis optic disc analysis comparing Cirrus spectral domain optical coherence tomography and Heidelberg retina tomograph II. Jpn J Ophthalmol.

[41] Weinreb RN (2004). Risk assessment in the management of patients with ocular hypertension. Am J Ophthalmol.

[42] Johnson CA (2000). The relationship between structural and functional alterations in glaucoma: a review. Semin Ophthalmol.

[43] Deleon-Ortega JE (2006). Discrimination between glaucomatous and nonglaucomatous eyes using quantitative imaging devices and subjective optic nerve head assessment. Invest Ophthalmol Vis Sci.

[44] Badalà F (2007). Optic disk and nerve fiber layer imaging to detect glaucoma. Am J Ophthalmol.

[45] Anderson D. R. & Patella V. M. *Automated Static Perimetry. 2nd ed*. Mosby (1999).

[46] Artes PH, Iwase A, Ohno Y, Kitazawa Y, Chauhan BC (2002). Properties of perimetric threshold estimates from Full Threshold, SITA Standard, and SITA Fast strategies. Invest Ophthalmol Vis Sci.

[47] Salvetat ML (2013). Learning effect and test-retest variability of pulsar perimetry. J Glaucoma.

[48] Bernardi L, Costa VP, Shiroma LO (2007). Flicker perimetry in healthy subjects: influence of age and gender, learning effect and short-term fluctuation. Arq Bras Oftalmol.

[49] Oleszczuk JD, Bergin C, Sharkawi E (2012). Comparative resilience of clinical perimetric tests to induced levels of intraocular straylight. Invest Ophthalmol Vis Sci.

[50] Matsumoto, C. *et al*. The influence of cataracts on perimetric threshold values in light-sense perimetry and flicker perimetry. *Perimetry**Update 2000/2001* (eds Wall M, Mills R, P.) Kugler 257 2001).

[51] Littmann H (1982). Determination of the real size of an object on the fundus of the living eye. Klin Monbl Augenheilkd.

